# A Sixteen-year Decline in Dissolved Oxygen in the Central California Current

**DOI:** 10.1038/s41598-018-25341-8

**Published:** 2018-05-08

**Authors:** Alice S. Ren, Fei Chai, Huijie Xue, David M. Anderson, Francisco P. Chavez

**Affiliations:** 1 School of Marine Sciences, University of Maine, Orono, Maine USA; 2 Scripps Institution of Oceanography, University of California, San Diego, La Jolla, California USA; 3 Monterey Bay Aquarium Research Institute, Moss Landing, California, USA

## Abstract

A potential consequence of climate change is global decrease in dissolved oxygen at depth in the oceans due to changes in the balance of ventilation, mixing, respiration, and photosynthesis. We present hydrographic cruise observations of declining dissolved oxygen collected along CalCOFI Line 66.7 (Line 67) off of Monterey Bay, in the Central California Current region, and investigate likely mechanisms. Between 1998 and 2013, dissolved oxygen decreased at the mean rate of 1.92 µmol kg^−1^ year^−1^ on σ_θ_ 26.6–26.8 kg m^−3^ isopycnals (250–400 m), translating to a 40% decline from initial concentrations. Two cores of elevated dissolved oxygen decline at 130 and 240 km from shore, which we suggest are a California Undercurrent and a California Current signal respectively, occurred on σ_θ_ ranges of 26.0–26.8 kg m^−3^ (100–400 m). A box model suggests that small annual changes in dissolved oxygen in source regions are sufficient to be the primary driver of the mid-depth declines. Variation in dissolved oxygen at the bottom of the surface mixed layer suggests that there is also a signal of increased local remineralization.

## Introduction

Oxygen in the world’s oceans is governed by three major processes: atmospheric exchange, ocean circulation, and the balance of respiration and photosynthesis^1^. Any shift in the balance of these three processes will disturb the mean dissolved oxygen concentration at a location. Indeed, among the many hypothesized impacts of climate change is a widespread decrease in mid-depth dissolved oxygen in the world’s ocean basins. The hypothesis suggests that increased surface heating and the resulting increased stratification of the oceans will decrease (1) the level of atmospheric exchange – less oxygen will dissolve into surface waters and (2) the mixing of surface waters to the deep ocean – less oxygen will reach deep waters^2–4^. It is unclear whether the climate change deoxygenation hypothesis is currently producing a measureable response from the ocean^5,6^ though Schmidtko *et al*.^7^ suggest that looking at the entire ocean oxygen inventory, there have been observed declines since the 1960s.

Interestingly, analysis of observational ocean data from the 1980s to the 2000s has suggested a decline in dissolved oxygen in the California Current region. Reports off of the Oregon and Washington coasts^8–10^ as well as reports in the Southern California Bight^11,12^ suggest declines in dissolved oxygen concentration over the roughly twenty-five-year period at up to 2.1 µmol kg^−1^ year^−1^ (Bograd *et al*.^12^ on a nearshore station in the Southern California Bight at 50 m). These declines could be related to observed decreases in dissolved oxygen in the North Pacific^13–18^ as well as in the tropical eastern Pacific, where observations suggest an expanding oxygen minimum zone^7,19^.

In one of the earlier attempts at explanation, Bograd *et al*.^12^ suggested that changing oxygen concentrations in the Southern California Bight could be caused by local increases in stratification due to surface heating, advection of low oxygen waters from the California Current (subarctic origin), advection of low oxygen waters from the California Undercurrent (tropical origin), or advection from the Subtropical Gyre. However, they stated that they were unable to discern whether the oxygen declines had been forced locally or forced remotely and subsequently advected into the region. Bograd *et al*.^11^ suggested that advection from the California Undercurrent and from the mean subtropical gyre circulation on the σ_θ_ = 26.5 kg m^−3^ surface are both important contributors of low oxygen water. Meinvielle and Johnson^20^ focused on the California Undercurrent, using σ_θ_ = 26.5 kg m^−3^ and bottom depths of 500–1000 m as the location of the undercurrent, and suggested that decreasing dissolved oxygen in the California Undercurrent is the cause of decreasing coastal dissolved oxygen. Pierce *et al*.^10^ studied the Newport hydrographic line and suggested that subarctic influence along σ_θ_ = 26.6 kg m^−3^, additional influence from the California Undercurrent on the slope, and shelf processes all contribute to observed decreases in dissolved oxygen. Focusing on shelf processes and secondarily on offshore dissolved oxygen declines, Connolly *et al*.^21^ suggested that shelf respiration processes are the primary cause of shelf hypoxia in the Northern California Current. It is likely that shelf hypoxia is related to the balance of shelf processes and the advection of offshore waters^22–24^, a balance which may be different from region to region^25,26^.

**Figure 1:**
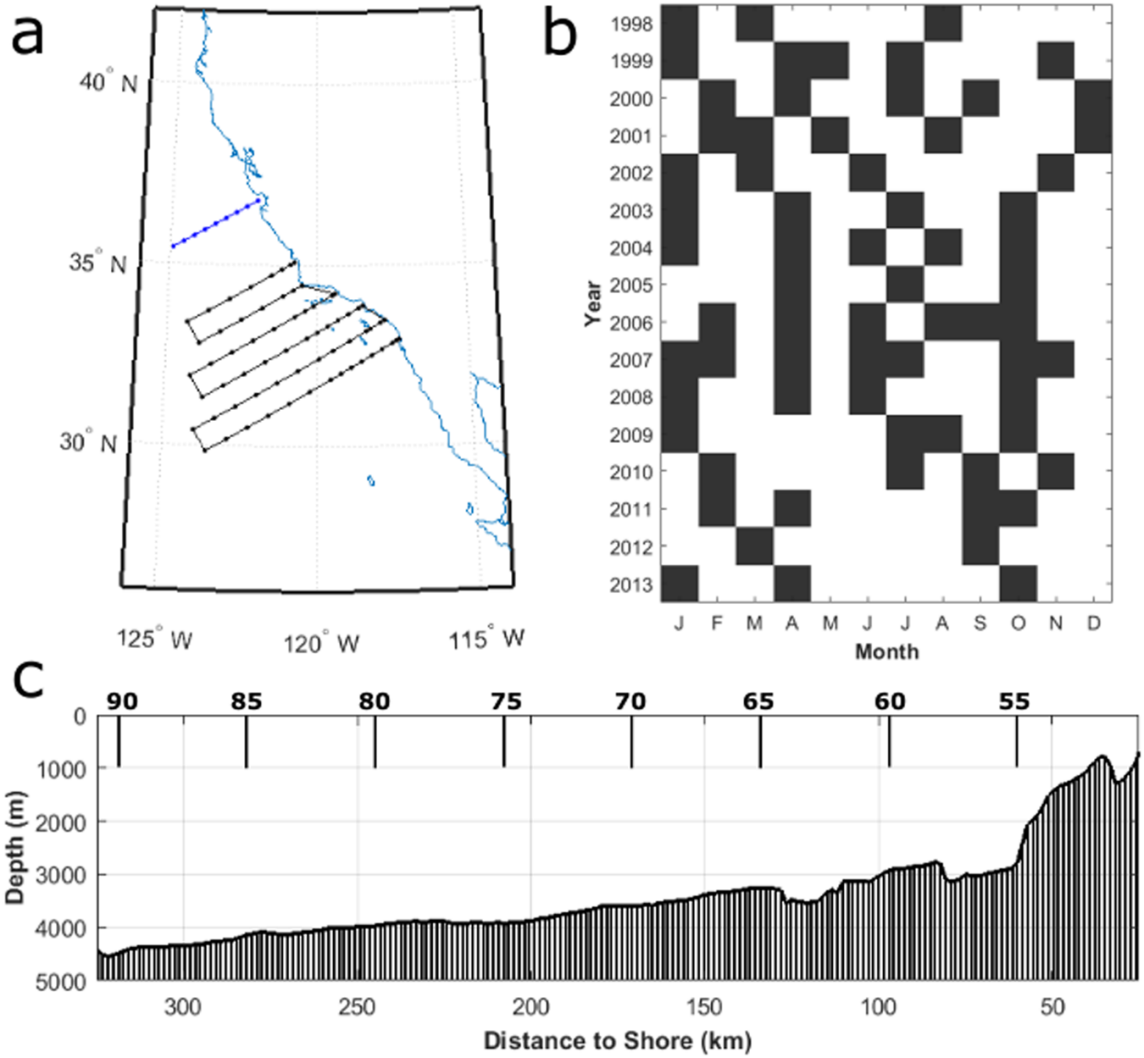
(**a**) Map of the location of CalCOFI Line 67, stations 50 to 90 (blue), and the standard CalCOFI sampling lines (black), (**b**) months of the year with Line 67 cruise data for dissolved oxygen on σ_θ_ = 26.7 kg m^−3^ and (**c**) bathymetry and location of Line 67 stations 55–90.

The purpose of this paper is to present the declining dissolved oxygen off of Monterey Bay, California, part of the Central California Current region, and to propose mechanisms to explain observed changes and interannual variability. Data for this analysis were at the same locations as CalCOFI Line 66.7 (Line 67), at stations 50, 55, 60, 65, 70, 75, 80, 85, and 90 but are independent of the CalCOFI data (Fig. 1a). Cruises occurred between 1 and 5 times per year, with the majority of years having more than 2 cruises (Fig. 1b). Line 67 does not represent a coastal shelf environment like the Newport hydrographic line in Oregon or parts of the Southern California Bight. Especially due to the proximity of Monterey Canyon, the coastal shelf is narrow, and there is a steep change in bathymetry close to shore (Fig. 1c). The hydrographic stations 65 to 90 sample in water of greater than 3000 m depth.

## Results

### Climatological dissolved oxygen and alongshore current structure

**Figure 2:**
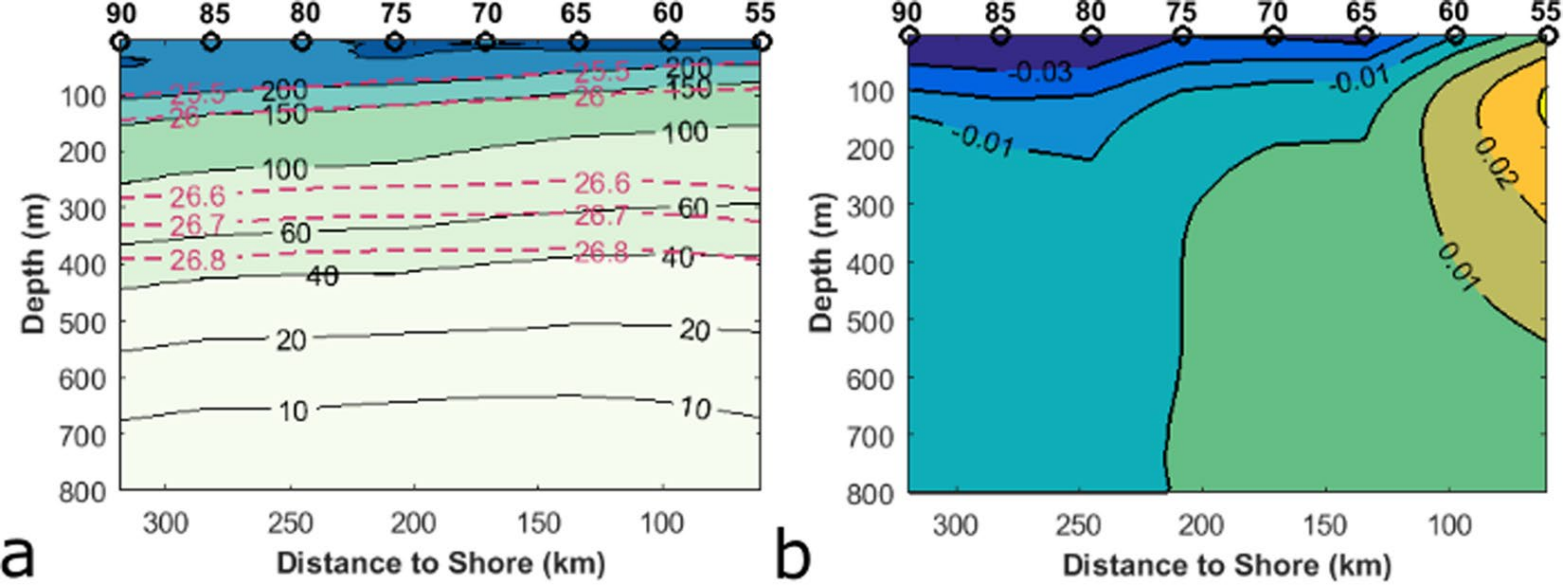
(**a**) Mean dissolved oxygen along Line 67 (µmol kg^−1^) and contours of potential densities 25.5, 26.0, 26.6, 26.7, and 26.8 kg m^−3^ (red, dashed). (**b**) Mean annual alongshore velocity at Line 67 (m/s). Positive values denote poleward alongshore flow. Black circles mark hydrographic stations.

The mean climatological dissolved oxygen level along Line 67 from 1998–2013 demonstrates that off of Monterey Bay the surface layer is well oxygenated while waters become hypoxic (defined as 60 µmol kg^−1^) at around 300–350 meters depth (Fig. 2a). Generally, especially in the upper water column, the surfaces of constant dissolved oxygen follow the surfaces of constant density. Below 150 m, the oxygen along an isopycnal is lower closer to shore and increases offshore.

The climatological alongshore velocity at Line 67 (Fig. 2b) demonstrates the poleward California Undercurrent as well as the equatorward California Current in the upper 200 m. The hydrographic data from station 50 were few, so station 50 was omitted, and the core of the California Undercurrent appears inshore of the plotted alongshore velocities. The division into a mean poleward regime and mean equatorward regime around 210 km is supported by other observational studies^27–29^. The demarcation around 100 km at the surface and 150 km at 200 m agrees with Collins *et al*.^29^. At around 300 m, the demarcation cannot be compared to Collins *et al*.^29^ who only calculated the velocities at the surface and 200 m. We did not find statistically significant trends in the alongshore velocity over time on any 5-meter averaged geostrophic velocity depth bin on each of the stations, nor did we find statistically significant trends in current transport over the top 200 m, 300 m, 400 m, or 500 m.

### Dissolved Oxygen Trends

**Figure 3:**
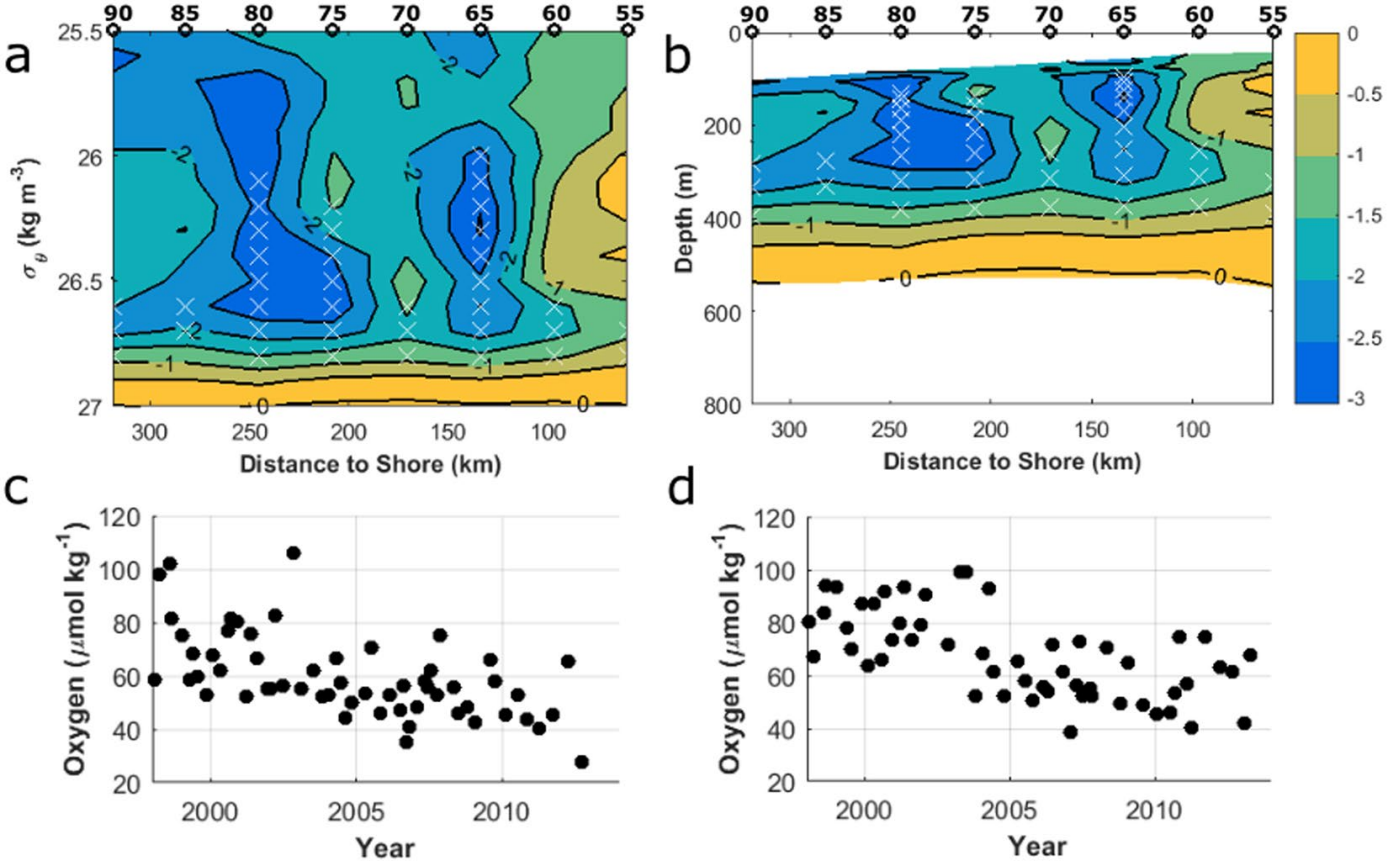
The rate of change in dissolved oxygen concentration (µmol kg^−1^ year^−1^) from 1998–2013 (**a**) on isopycnals (σ_θ_ 25.5–27.0 kg m^−3^) and (**b**) on depth. The rate of change was calculated along isopycnals and is plotted over the average depth of each σ_θ_ from 25.5–27.0 kg m^−3^. In both, white crosses mark statistically significant correlations (p < 0.01, corrected for autocorrelation) while black circles mark hydrographic stations. (**c**) The observations of dissolved oxygen on σ_θ_ 26.7 at station 65 and (d) the observations of dissolved oxygen on σ_θ_ 26.7 at station 80.

Decreases in dissolved oxygen occurred on σ_θ_ 26.6–26.8 kg m^−3^ for almost all stations (Fig. 3a) and ranged from −0.67 to −2.90 µmol kg^−1^ year^−1^. For stations 65, 75, and 80 linear declines in dissolved oxygen were also found between σ_θ_ 26.0–26.6 kg m^−3^. Very strong linear declines (−2.54 to −2.91 µmol kg^−1^ year^−1^) were found from σ_θ_ 26.1 to 26.5 kg m^−3^ on station 80, roughly 240 km from shore and from 26.0 to 26.5 kg m^−3^ on station 65, roughly 130 km from shore (−2.41 to −3.06 µmol kg^−1^ year^−1^). Mapped from density to depth coordinates (Fig. 3b), there is an area of strong declining dissolved oxygen between 200 and 250 km from shore at 125–300 m, around 130 km from shore at 100–300 m, and through the entire transect at depths of 300–400 m.

**Figure 4:**
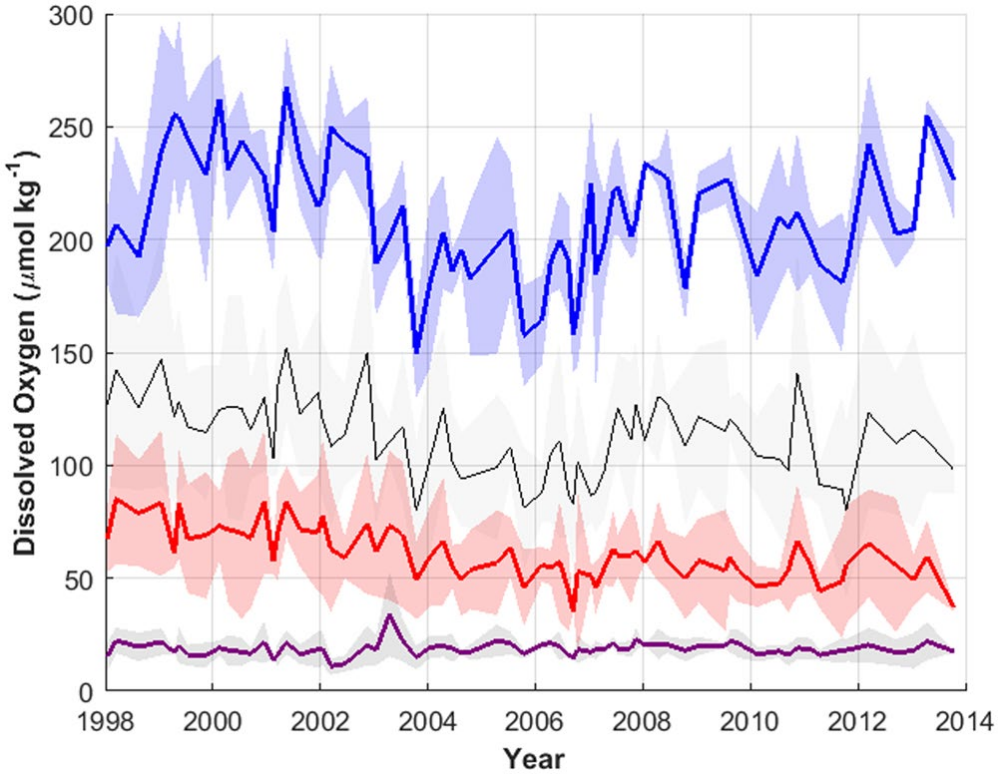
Line 67 mean dissolved oxygen concentrations along σ_θ_ 25.5 kg m^−3^ (blue), 26.3 kg m^−3^ (black), 26.7 kg m^−3^ (red), 27.0 kg m^−3^ (purple) from 1998 to 2013. Shaded regions represent two standard deviations from the mean.

There are differences in the oxygen dynamics along isopycnals closer or farther from the ocean surface (Fig. 4). On the 25.5 kg m^−3^ σ_θ_ surface, found at roughly 50–100 m depth, the dissolved oxygen concentrations have stronger inter-annual variability and potentially exhibit a low-frequency oscillatory pattern with a period of less than 10 years. At and below the 26.1 kg m^−3^ σ_θ_ surface, at roughly 100–150 m depth, a statistically significant decline exists (p = 0.00003, corrected for autocorrelation), though there continues to be low frequency inter-annual variability. On the 26.7 kg m^−3^ σ_θ_ surface, found at roughly 300–350 m depth, the year-to-year variability is less, and over 1998–2013 there is a linear decline in dissolved oxygen. While at σ_θ_ = 26.7 kg m^−3^ the stations closer to shore had lower dissolved oxygen, a similar trend was found in all stations (not shown), justifying the presentation of the dynamics of σ_θ_ = 26.7 kg m^−3^ as the average over all the stations of the transect. On σ_θ_ = 25.5 kg m^−3^, there was no significant difference in the dissolved oxygen concentration between stations.

The linear declining trend disappears for σ_θ_ ranging from 26.9 to 27.3 kg m^−3^, within which there appears to be no, or very little, temporal trend in the oxygen concentration. From 27.3–27.4 kg m^−3^, there is a small positive temporal trend in dissolved oxygen (0.39 µmol kg^−1^ year^−1^, p = 1 × 10^−12^). Data availability for dissolved oxygen is low below 1000 m or around σ_θ_ 27.4 kg m^−3^. Deep casts below 1000 m were not made with sufficiently high frequency to investigate a temporal trend.

Declines along σ_θ_ 26.7 kg m^−3^ are in areas just above the hypoxic boundary (60 µmol kg^−1^). In 1998, the averaged oxygen concentration at σ_θ_ 26.7 kg m^−3^ was 76.9 µmol kg^−1^, and the average rate of decline was 1.92 µmol kg^−1^ year^−1^. Thus, the average station oxygen concentration at the end of 2013 according to the linear model was 46.2 µmol kg^−1^, representing a 39.9% loss in dissolved oxygen from initial concentrations along σ_θ_ 26.7 kg m^−3^. The decline in oxygen concentration occurred without statistically significant changes in the depth of σ_θ_ 26.7 kg m^−3^.

### Mid-depth decreases in dissolved oxygen

**Figure 5:**
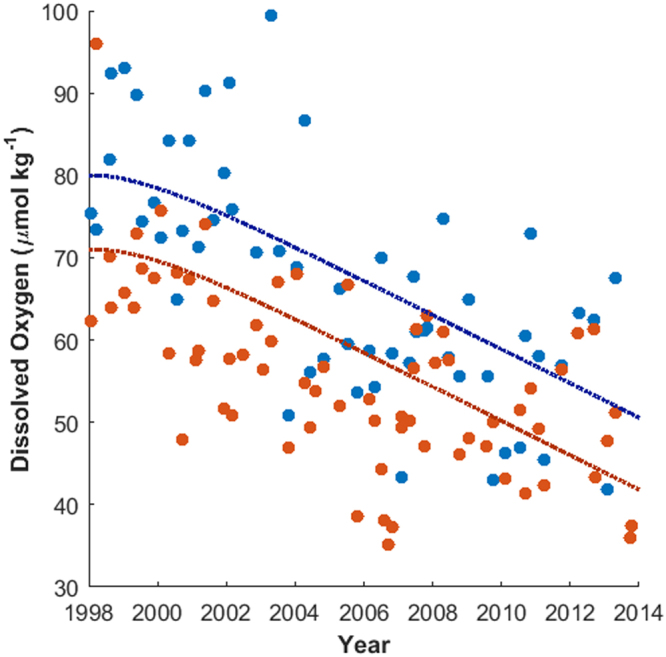
Inshore (average of stations 55–65, orange circles) and offshore (average of stations 75–85, blue circles) dissolved oxygen from Line 67 observations over time along σ_θ_ = 26.7 kg m^−3^. Box model dissolved oxygen over the first 16 years of simulation are plotted for the inshore (orange line) and offshore (blue line) boxes. The simulation used annual decline in northern and southern source water dissolved oxygen of 2.08 µmol kg^−1^ year^−1^. Linear rates of decline over the time period in the box model are 1.94 µmol kg^−1^ year^−1^ and 1.92 µmol kg^−1^ year^−1^ for the inshore box and offshore box respectively.

The declines in dissolved oxygen concentration from 1998–2013 found in the Line 67 data occurred in two cores between σ_θ_ 26.0–26.6 kg m^−3^ and along σ_θ_ 26.7 kg m^−3^ across the entire 300-km transect (Fig. 3). There was oxygen decline in both directions of alongshore current; thus we set up a box model consisting of an inshore box with the California Undercurrent and an offshore box with the California Current (Supplementary Fig. S1). In the box model, we included the local respiration, the alongshore currents, and their source waters. We found little change in observed density structure along Line 67 over time, so we did not include stratification. We used the box model to reproduce the declines in dissolved oxygen along Line 67 (Fig. 5) on σ_θ_ 26.7 kg m^−3^ for both the inshore, undercurrent region and the offshore, California Current region.

The box model was run for a 20-year simulation period to test three mechanisms: the source water oxygen concentration, the strength of the alongshore transport, and the local respiration. In order for the local respiration to be solely responsible for the decline in dissolved oxygen at the rate of over 1.92 µmol kg^−1^ year^−1^, the respiration needed to increase from 2.41 µmol kg^−1^ year^−1^ to 25.41 µmol kg^−1^ year^−1^ in the offshore box, and from 1.32 µmol kg^−1^ year^−1^ to 23.32 µmol kg^−1^ year^−1^ in the inshore box over the time period. In order for the strength of the alongshore transport to be solely responsible for the decline in dissolved oxygen, the strength of the poleward alongshore transport needed to increase by over 30 times (from 0.01 m/s to 0.30 m/s), and the equatorward transport needed to stop (0.01 m/s to 0 m/s) over the time period. The result of this perturbation scenario was a 1.62 µmol kg^−1^ year^−1^ decrease in the offshore, California Current-controlled, box and a 1.08 µmol kg^−1^ year^−1^ decrease in the inshore, California Undercurrent-controlled, box. The simple box model was not able to achieve steady state with larger perturbations in the alongshore transport. In order for the source water oxygen concentration to be solely responsible for the decline, the concentrations of both source waters would need to decline by 2.08 µmol kg^−1^ year^−1^ over the time period.

With these comparisons, the most realistic cause for a large decrease in dissolved oxygen was a decrease in the oxygen concentration of the source waters (Fig. 5). The rate of dissolved oxygen decline in the box model is similar to but somewhat higher than that of reported dissolved oxygen declines. While a decline of 2.08 µmol kg^−1^ year^−1^ is on the high end of the 0.9–1.7 µmol kg^−1^ year^−1^ decline reported in the Southern California Bight^11^, it is more than twice as high as the upper rate of decline estimated from the Newport, Oregon data at 0.9 µmol kg^−1^ year^−1^ ^10^. If the northern source water dissolved oxygen decline is set to 0.9 µmol kg^−1^ year^−1^ and the southern source water dissolved oxygen decline remains at 2.08 µmol kg^−1^ year^−1^, the rate of decline is 1.19 µmol kg^−1^ year^−1^ in the offshore box and 1.40 µmol kg^−1^ year^−1^ in the inshore box, representing 62% and 73% of the observed rate of decline, respectively. The remaining 38% and 27% of dissolved oxygen decline could be caused by additional mechanisms such as the local respiration. It is highly plausible that multiple mechanisms are at work.

In summary, the primary reason for observed mid-depth declines in oxygen on Line 67 appears to be remote forcing, and the perturbations need to occur in both source waters, one feeding the California Current and one feeding the California Undercurrent.

### Upper Level Oxygen Variability

The dissolved oxygen concentrations on upper level isopycnals exhibit large interannual fluctuations that are likely caused by natural climate variability. Correlation of annual values of an oxygen index created by normalizing observed concentrations by their standard deviation was significant with the North Pacific Gyre Oscillation (NPGO) (R = 0.72, p = 0.002, no significant autocorrelation) and the Upwelling Index (UI) (R = 0.63, p = 0.009, no significant autocorrelation). In particular, the correlation of the oxygen index and UI in spring was high (R = 0.71, p = 0.004, see Supplementary Fig. S2). The oxygen index was not significantly correlated with the Oceanic Nino Index (ONI) (R = −0.43, p = 0.10, no significant autocorrelation).

**Figure 6:**
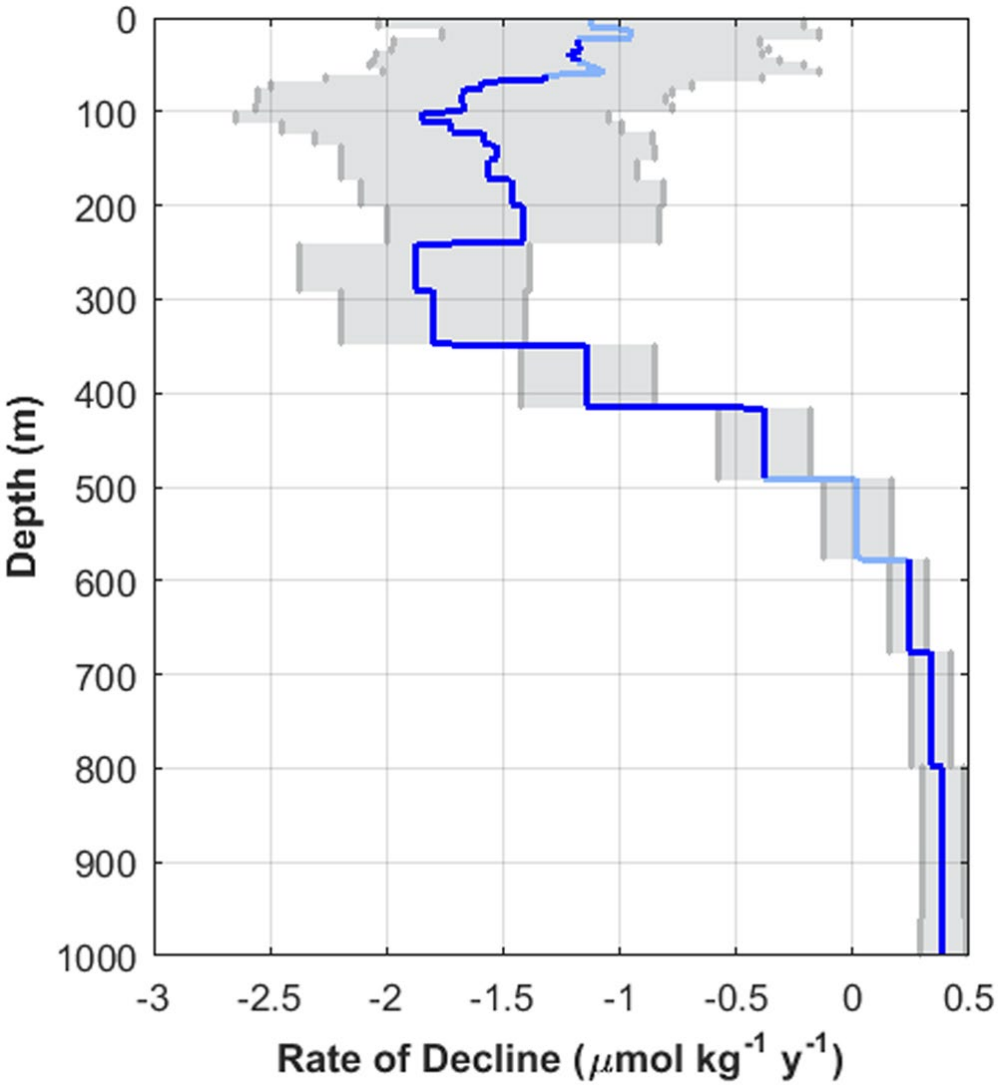
Depth profile of the rate of decline of dissolved oxygen concentration (µmol kg^−1^ year^−1^) on Line 67 (blue line). The 95% confidence level of the rate of decline is shaded in gray. Rates of change that are not significant (p > 0.01, Student’s t-test) are plotted in light blue.

In addition to the variability above 300 m, there are significant declines in dissolved oxygen (Fig. 6). The vertical profile of declining dissolved oxygen, calculated by finding the average potential density on each depth and reporting the rate of decline as calculated on that potential density, suggests that there is a declining oxygen trend with a peak around 100 m in addition to the peak around 300 m associated with σ_θ_ 26.7. The shallow peak is just beneath the pycnocline and is what would be expected from increased remineralization due to increased primary production which has been documented off of Monterey Bay^30^. The decrease and subsequent increase in the rate of decline supports the possibility of two different mechanisms. We suggest that the deeper peak is indicative of decreased dissolved oxygen transport from changing large-scale ventilation and that the shallower peak is indicative of increased remineralization from increased local primary production.

## Discussion

The results of the time series along Line 67 off of Monterey Bay along with existing published results suggest that there is significant decline in dissolved oxygen occurring in the northern, central, and southern California Current regions, which suggests that deoxygenation is part of a large-scale phenomenon across the California Current system (Table 1). The mean rate of decline along Line 67, at σ_θ_ 26.7 kg m^−3^ density surface, is comparable to but larger than the rate in the Southern California Bight. Reported declines off of Oregon^10^ and in the North Pacific at Ocean Station Papa^18^ have been at rates less than 1 µmol kg^−1^ year^−1^.

**Table 1:** Summary and comparison of rates of decline in the California Current and Northeast Pacific.

Researcher	Density	Decline (µmol kg^−1^ year^−1^)	Time Period	Region
Bograd *et al*.^11^	26.5	**−0.87 to −1.7** ^**†**^	1984–2012	S. California Bight
Pierce *et al*.^10^	26.3–26.5 26.7	**−0.24 to −0.86°** **−0.27 to −0.41°**	1960s–2000s	Newport, Oregon Line
Whitney *et al*.^18^	26.3–27.0	**−0.4 to −0.7**	1950s–2006	Ocean Station Papa (50°N, 145°W)
This Study	26.7	**−1.92**	1998–2013	Line 67, Central California

^†^Derived from Fig. 3 in Bograd *et al*.^11^. °Taken from Table 1 in Pierce *et al*.^10^.

While observations of decline off of Central California reinforce other reports of ocean deoxygenation in recent decades, additional studies are needed to determine whether climate change or natural variability is the underlying cause. However, we do describe in more detail the mechanisms governing deoxygenation, especially in the California Current region, and especially their relationship to large-scale changes in Pacific Ocean biogeochemistry.

In terms of source water changes, we suggest that there may be two different processes at work within the Pacific Basin, both of whose signatures we find in the California Current System. Deutsch *et al*.^5^ suggested that the thermocline depth in the tropics drives the expansion or contraction of the volume of hypoxic waters in the tropical Pacific, with a shallower thermocline causing increased respiration and the expansion of the oxygen minimum zone. The low oxygen waters from the tropics could then be transported via the California Undercurrent poleward to affect the California Current region. On the other hand, analysis of Station Papa at 50°N 145°W^18^, the CLIVAR and WOCE transects at 152°W^17^, and observations in the western North Pacific^14^ suggested that ventilation of σ_θ_ 26.6 kg m^−3^ periodically ceases, causing decreased dissolved oxygen concentrations that subsequently spread throughout the subtropical gyre along σ_θ_ 26.6 kg m^−3^. Kwon *et al*.^31^ studied central mode waters in the North Pacific defined on neutral densities of 25.6–26.6 kg m^−3^ and suggested that the area through which the oxygen-rich mixed layer is detrained into the thermocline varies decadally, with a connection to the Pacific Decadal Oscillation (PDO). Waters with a low-oxygen anomaly formed in the North Pacific could be transported to the California Current region in the California Current or the mean subtropical gyre circulation. Additional modeling studies could test our hypothesis. Finally, such decreases in oxygen concentration from large-scale currents in the coastal upwelling region have the potential to exacerbate shelf hypoxia.

## Data and Methods

### Data Collection and Processing

Quasi-seasonal cruise data were collected from 1998 to 2013 as part of various projects of the Biological Oceanography Group (BOG) at the Monterey Bay Aquarium Research Institute (MBARI). Dissolved oxygen data were taken with an Aanderaa optode oxygen sensor. The sensor data were averaged on 1-meter vertical depth bins. The horizontal resolution of the data was dictated by the distance between stations along Line 67, roughly 40 km. The oxygen sensor data for each cruise were calibrated with dissolved oxygen bottle samples analyzed with a Winkler titration. Temperature and salinity observations were recorded with a CTD. Values were also averaged to 1-meter vertical depth bins.

The collection of cast data contained 590,337 sensor observations. Quality control of the data was as follows. First, data greater than 10 ml/l and at or below 0 ml/l were removed from the analysis because they were outside the normal range of oxygen values for the region. Second, the potential density of all observations was calculated from the observed *in-situ* temperature and salinity, and all oxygen observations for which there were no temperature and salinity readings, or for which the temperature or salinity cast profile was deemed suspect, were removed from the analysis. In a few cases, instead of the primary temperature and salinity observations, the temperature and salinity measurements from a secondary CTD with a more realistic profile were used. Roughly 3% of the data were removed from analysis in these steps, a total of 15,586 oxygen sensor observations. Finally, all values of oxygen were calculated in µmol kg^−1^ using the molar volume of oxygen at STP of 22.3916 L/mole and the calculated potential density. One ml/L is equivalent to 44.6596 µmol/L. To display the variability in dissolved oxygen over time, monthly averages of dissolved oxygen values were used, where data were first binned and averaged for each day (if repeat casts were taken), then month. Months without data in the time-series were noted.

### Alongshore Velocity

*In-situ* temperature and salinity were used to calculate alongshore geostrophic velocities. Temperature and salinity observations were binned to 5-meter depth intervals and by station. Geostrophic velocities were calculated with a reference depth of 800 meters. The reference depth was chosen as a compromise between depth and the availability of temperature and salinity observations. It is possible that there exists a level of motion at 800 meters depth, in which case the relative patterns remain valid. The CalCOFI lines run approximately normal to the coastline, so we considered the cross-transect direction to be alongshore. At Line 67, alongshore phenomena are roughly a coordinate rotation counterclockwise of 30 degrees.

### Bathymetry

Data to plot the bathymetry of Line 67 came from two sources. Inshore of station 60 the bathymetry is from the NOAA National Geophysical Data Center 3 Arc-Second Coastal Relief Model retrieved from the Southern California Coastal Ocean Observing System at http://sccoos.org/data/bathy. Offshore of station 60 the bathymetry is from the General Bathymetric Chart of the Oceans (GEBCO) GEBCO_2014 Grid, version 20150318, retrieved from http://www.gebco.net.

### Dissolved Oxygen Trends

To more clearly understand ocean mixing processes and to remove the influence of heave, or isopycnal motions with no heat and salinity exchange with the environment (as defined in Huang^32^, although the term originates much earlier), oxygen variability was analyzed along potential density surfaces. For time series of data along a potential density surface, we took into account variability due to the different stations along the transect (over 200 km long) and seasonality. With regard to transect stations, a chi-squared test for independence was used to determine if the oxygen values for each density were independent of the station number.

To investigate spatial patterns of the rate of change in dissolved oxygen, changes in time of oxygen concentration were assessed at observations binned to 0.1 kg m^−3^ σ_θ_ and by station. Station 50 was removed from analysis due to low number of observations. The correlation (R-value), two-tailed significance of the correlation (p), and the constants for a linear model (mx + b, where time was the x-variable) were calculated for each bin. We used anomalies to calculate the correlation and significance, subtracting seasonal averages from monthly averaged observations. To map the calculations back to coordinates for depth and distance offshore, statistics along isopycnals were mapped to the average depth of each isopycnal in the analysis over the data collection period. To create the vertical profile of rates of decline, rate of decline was calculated with a linear regression model of the oxygen data at each bin of 0.1 kg m^−3^ σ_θ_. The profile displays the rate of decline as calculated on the average potential density found at each depth. We tested the significance with the Student’s t-test for the slope of a linear regression model.

### Climate Index Correlations

To investigate potential drivers for oxygen dynamics, oxygen time-series on specific potential densities were compared to climate indices important for ocean dynamics in the Pacific Ocean with variations on the timescale of 4–10 years using correlation. To represent the El Nino oscillation, we used the Oceanic Nino Index (ONI), which is the three-month running mean of temperature anomalies from the Nino-3.4 region (5°S to 5°N; 170°W to 120°W). To represent the North Pacific Gyre Oscillation (NPGO) we used the NPGO index downloaded from http://www.o3d.org/npgo/ and updated on January 2016. The NPGO is the second mode of sea surface height variability in the North Pacific which is related to the strength of the subtropical gyre circulation^33^. To represent local upwelling, we used the NOAA PFEL upwelling index for the period 1998–2013 at 36°S 122°W. The index is calculated from the geostrophic wind stress which is derived from sea level pressure from the U.S. Navy Fleet Numerical Meteorology and Oceanography Center’s operational forecasts. We used the monthly mean anomalies downloaded from http://www.pfeg.noaa.gov/products/PFELData/upwell/monthly/upanoms.mon.

To compare climate index and dissolved oxygen variability, variation in indices and oxygen time-series were normalized by standard deviations and compared on an annual basis. The annual means used the average of the normalized monthly anomalies for dissolved oxygen observations and the average of the normalized monthly or seasonal values for the climate indices. In addition, seasonal time-series were defined in three-month intervals with winter as December through February.

### Autocorrelation

A major complication in correlation of geophysical and climate phenomena is autocorrelation of time-series data^34,35^. Consequently, all correlations were tested for lag-1 autocorrelation. If significant autocorrelation were detected, the true significance of the correlation was corrected by using an effective sample size when calculating the t-statistic and as the sample size to calculate the p-value^36^. The effective sample size, n_e_, is defined according to the lag-1 autocorrelation coefficient r_1_ and the original sample size n by:

1 $${{n}}_{{e}}\approx {n}\frac{1-{r}_{1}}{1+{r}_{1}}.$$

### Box Model

We attempted to better understand why the dissolved oxygen in Central California had declined along Line 67 using a box model to estimate local respiration, source water dissolved oxygen concentration changes, and changes in the magnitude of currents acting in concert. The initial concentrations of oxygen in nearshore and offshore boxes and the initial alongshore geostrophic velocity were taken from Line 67 observations. Initial concentrations of source waters in dissolved oxygen and salinity were taken from the World Ocean Atlas (WOA) 2013. The gradients in the model were balanced at steady state using salinity; we adjusted the source water salinity by a correction factor and used an empirical relationship between salinity and oxygen^37^ to subsequently adjust the source water oxygen. Additional information on the setup of the box model is provided in the supplementary materials.

### Data availability

The datasets analyzed during the current study are available from the corresponding author on reasonable request.

## Electronic supplementary material

Supplementary Information
