# Supplementary material for: A Sixteen-year Decline in Dissolved Oxygen in the Central California Current
Source: Sci Rep. 2018 May 8;8:7290. doi: 10.1038/s41598-018-25341-8 (PMC5940839; doi:10.1038/s41598-018-25341-8)
Supplement: Supplementary file 1 — Supplementary Information [file 41598_2018_25341_MOESM1_ESM.pdf]

A Sixteen-year Decline in Dissolved Oxygen in the Central California Current

Alice S. Ren[1,2]*, Fei Chai[1], Huijie Xue[1], David M. Anderson[3], Francisco P. Chavez[3]

1, School of Marine Sciences, University of Maine, Orono, Maine, USA

2, Scripps Institution of Oceanography, University of California, San Diego, La Jolla, California, USA

3, Monterey Bay Aquarium Research Institute, Moss Landing, California, USA

Corresponding author: Alice S. Ren

Address: Scripps Institution of Oceanography, UC San Diego, 9500 Gilman Drive, La Jolla, CA 92093

Email: aren@ucsd.edu

Supplementary Materials

Box Model. The box model was set up with two boxes, one to represent the equatorward California Current and one to represent the poleward California Undercurrent (Supplementary Fig. S1). The alongshore transport from the California Current brings a net import of dissolved oxygen into the offshore box, due to the gradient set up by the oxygenated northern source waters versus the Line 67 waters. The alongshore transport from the California Undercurrent results in a net loss of dissolved oxygen in the inshore box, due to the gradient set up by the less oxygenated southern source waters compared to the Line 67 waters. At equilibrium, the oxygen concentrations of both inshore and offshore boxes should not change. In each box, cross-shore mixing and local respiration act to balance the advective inputs or losses of oxygen. The box model parameters are summarized in Supplementary Table 1.

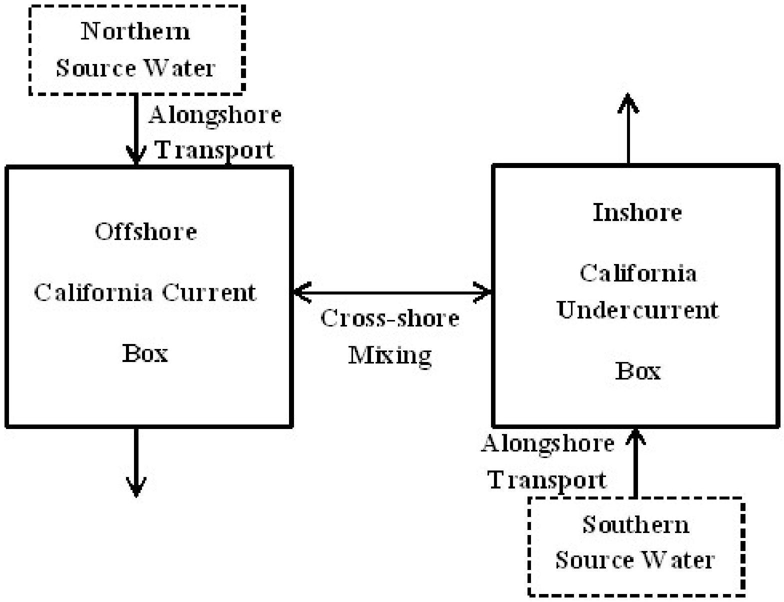

Supplementary Figure S1. Schematic of Central California Current box model.

Boxes were located at the depth of greatest dissolved oxygen decline, which was around 300 m depth and σ_θ 26.7 kg m^{-3}. The box size was chosen to represent a slice of ocean with a thickness of 100 m and a width of 50 km. The alongshore box length was chosen from the distance a particle would travel at the alongshore current speed within one model time step.

The initial concentrations of source waters in dissolved oxygen and salinity were taken from the World Ocean Atlas (WOA) 2013, where climatological properties are calculated on a 1°x1° grid. The source water locations were, for the northern source water, at 39.5 °N, 127.5 °W and, for the southern source water, at 31.5 °N, 117.5 °W. The southern source water is off Baja California and was chosen to be farther from the region to avoid sampling near the complex circulation of the Southern California Bight. The initial concentrations of dissolved oxygen in the inshore and offshore boxes (representative of Line 67) were taken from the average inshore (stations 55-65) and offshore (stations 75-85) station oxygen concentrations in roughly 1999. The choice was also made because linear regression models of station 80 and station 65 oxygen data differed by roughly 9 µmol/kg at the start of the time period.

Average alongshore current speeds were those from the calculated geostrophic currents along Line 67. The cross-shore mixing was calculated using a one-dimensional Fick's law formulation with a mixing coefficient, D. The mixing coefficient, D, was calculated using a salinity balance in the box model. The salinity gradients were determined by the difference between source water and Line 67 box concentrations. At steady state, the salt transported into, mixed, and transported out of each box should balance. As the WOA data were of coarse resolution, we corrected for a steady state imbalance in the box model by adjusting the source water salinity by a correction factor. Consequently, the oxygen of the source waters was also adjusted by a correction factor (Supplementary Table 1). The change of salinity was converted into a change in dissolved oxygen according to the linear relationship found in *Castro et al.* [2001]. The oxygen gradients were determined by the difference between source water and Line 67 box concentrations. Respiration in the two boxes was calculated from the steady state solution of the box model knowing the cross-shore mixing and alongshore fluxes of oxygen. As a consequence of being at depth, the biological component was assumed to be only respiration.

Supplementary Table 1. Box Model Parameters

Parameter	Value
**Box height**	100 m
**Box width (cross-shore direction)**	50 km
**Box length (alongshore direction)**	78.8 km
**Box depth**	250-350 m
**Offshore box alongshore velocity**	0.01 m/s (equatorward)
**Inshore box alongshore velocity**	0.01 m/s (poleward)
**Model time step**	0.25 years
**Northern source water oxygen**	100. µmol/kg
**Northern source water oxygen (corrected)**	100.3 µmol/kg
**Southern source water oxygen**	49.5 µmol/kg
**Southern source water oxygen (corrected)**	49.9 µmol/kg
**Northern source water salinity**	34.006
**Northern source water salinity (corrected)**	34.000
**Southern source water salinity**	34.235
**Southern source water salinity (corrected)**	34.225
**Northern source water distance (to Line 67, station 80)**	505 km
**Southern source water distance (to Line 67, station 65)**	743 km
**Initial dissolved oxygen of inshore box**	71 µmol/kg
**Initial dissolved oxygen of offshore box**	80 µmol/kg
**D**	199 m^2/s
**Cross-shore distance between boxes**	110 km

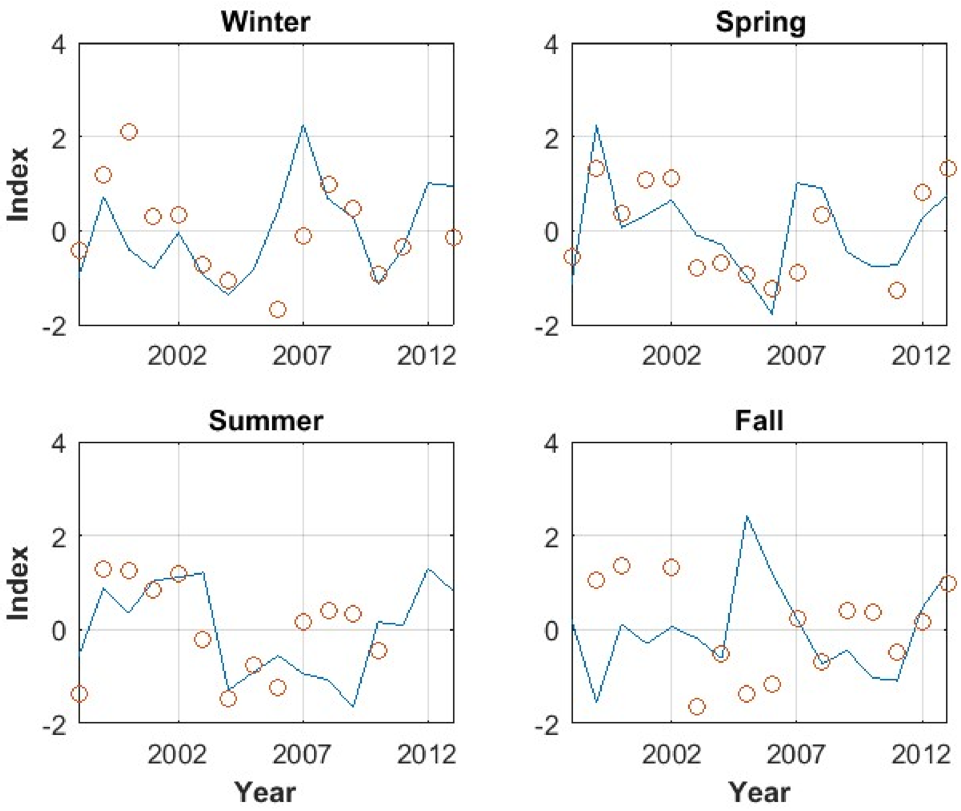

Supplementary Figure S2. Comparison of seasonal anomalies of dissolved oxygen (circles, orange) on σ_θ 25.5 kg m^{-3} and seasonal upwelling index anomalies (solid line, blue) for winter (R = 0.24, p = 0.4), spring (R = 0.71, p = 0.004), summer (R = 0.52, p = 0.07), and fall (R = -0.27, p = 0.3). Seasons are defined in three-month intervals with winter defined as December-February.
